# DFT modeling of water-assisted hydrogen peroxide formation from a C(4a)-(hydro)peroxyflavin

**DOI:** 10.55730/1300-0527.3673

**Published:** 2024-04-18

**Authors:** Yılmaz ÖZKILIÇ

**Affiliations:** Department of Chemistry, Faculty of Science and Letters, İstanbul Technical University, İstanbul, Turkiye

**Keywords:** Hydrogen peroxide, DFT, mechanism, C(4a)-(hydro)peroxide, uncoupling, kynurenine 3-monooxygenase

## Abstract

The cofactor of a class A monooxygenase is reduced at an external location of the enzyme and is subsequently pulled back into the active site after the reduction. This observation brings the question; is there any defense mechanism of the active site of a monooxygenase against the formation of the harmful hydrogen peroxide from the reactive C(4a)-(hydro)peroxide intermediate? In this study, the barrier energies of one to three water molecule-mediated uncoupling reaction mechanisms in water exposed reaction conditions were determined. These were found to be facile barriers. Secondly, uncoupling was modeled in the active site of kynurenine 3-monooxygenase complex which was represented with 258 atoms utilizing cluster approach. Comparison of the barrier energy of the cluster model to the models that represent the water exposed conditions revealed that the enzyme does not have an inhibitory reaction site architecture as the compared barrier energies are roughly the same. The main defense mechanism of KMO against the formation of the hydrogen peroxide is deduced to be the insulation, and without this insulation, the monooxygenation would not take place as the barrier height of the hydrogen peroxide formation within the active site is almost half of that of the monooxygenation.

## 1. Introduction

**Figure 1:**
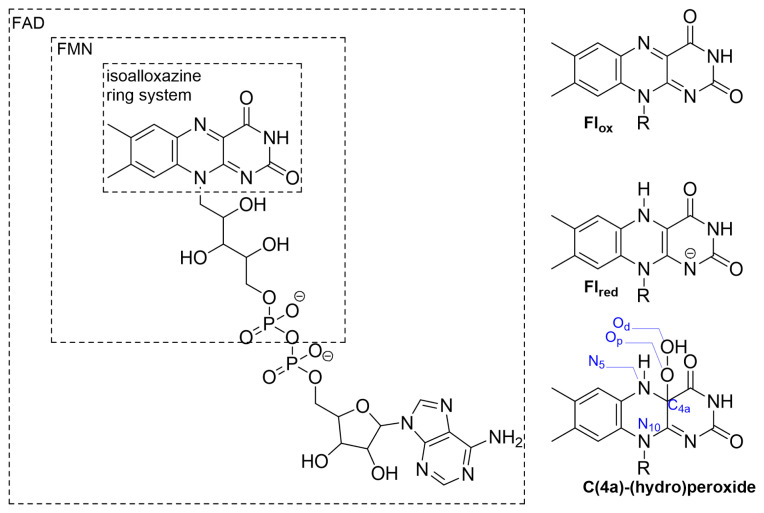
Flavin mononucleotide (FMN) and flavin adenine dinucleotide (FAD) structures with their different oxidation states [2].

Flavoenzymes are various enzymes such as dehydrogenases, reductases, oxidases and monooxygenases [1–4]. A flavoenzyme’s activity strictly depends on an organic cofactor (Figure 1) that is found in two varieties: a flavin mononucleotide (FMN) or a flavin adenine dinucleotide (FAD) [4]. Isoalloxazine ring system of these cofactors is the chemically involved moiety (Figure 1) that takes part in the activation processes, and the remaining part of the molecule holds the cofactor in an interior location of the protein so that the reactions can take place in a buried environment that prevents the disruptive effects of other molecules; especially the solvent water [5].

Oxidases and monooxygenases are responsible for the activation of the molecular oxygen, which in its triplet ground state cannot spontaneously participate in most of the chemical reactions [2]. According to experimental and computational studies, the flavoenzyme catalyzed oxygen activation mechanisms generally involve two half-reactions in which the isoalloxazine ring system is reduced and oxidized consecutively (Figure 1) [6–28]. During the reduction of the class A monooxygenases, FAD changes its conformational state to meet with the large reducing agent NADPH at an outer location of the enzyme then immediately returns to its original conformational state [5]. The details and classifications of these reactions are nicely explained in recent reviews [1–3].

**Figure 2:**
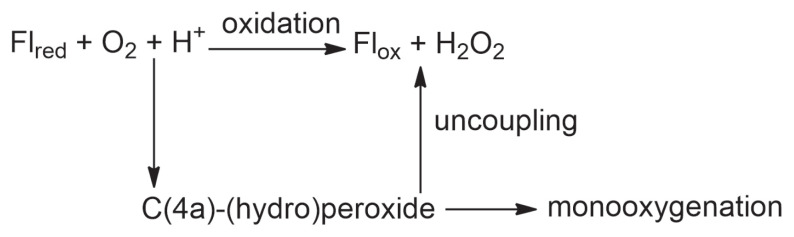
Concise summary of the conventional reactions between reduced flavins (Fl_red_) and molecular oxygen [2, 11].

The prime objective of the present study is to understand how monooxygenases act like oxidases. In Figure 2, the reactions between reduced flavins and the molecular oxygen are concisely summarized for the oxidases and monooxygenases. Oxidases catalyze the formation of hydrogen peroxide (H_2_O_2_), while monooxygenases catalyze the formation of an oxygen adduct of a substrate through the reactive C(4a)-(hydro)peroxide intermediate whose one of the oxygen atoms is transferred to the substrate [2]. When this substrate is not present in the active site of a monooxygenase, an event known as uncoupling takes place: hydrogen peroxide is formed through the oxidation of the flavin (Figure 2) [2]. Interestingly, uncoupling can take place even when the substrate is within the active site [29]. Hydrogen peroxide accelerates aging, cancer cell metabolism, and inflammation [30]. In addition, hydrogen peroxide increases oxidative stress in neurons which induces neuronal cell death and thereby can trigger a variety of neurodegenerative diseases [31,32]. Generation of hydrogen peroxide in a monooxygenase can complicate therapeutic strategies. An infamous example is the inhibition of kynurenine 3-monooxygenase (KMO) whose product’s (3-hydroxykynurenine: 3-HK) overproduction is known to cause several neurodegenerative diseases [33,34]. Most KMO inhibitors successfully inhibit the formation of 3-HK and thereby the monooxygenation of the substrate L-kynurenine (L-Kyn). However, they can also cause the reduction of Fl_ox_ which leads to the formation of the unstable C(4a)-(hydro)peroxide intermediate that inevitably relaxes with uncoupling [29,35,36]. Therefore, the struggle to prevent a kind of neurodegenerative disease inducer leads to another due to the complication introduced by the generation of hydrogen peroxide as a side product.

According to mechanistic studies, the monooxygenation reaction can take place with lower barrier energies when it is taking place within the active site of a monooxygenase. However, the barrier energy lowering offered by the enzyme is not significant, meaning that the reaction between the C(4a)-(hydro)peroxide and the substrate would still take place if only the disruptive effect of the environment was circumvented [16,20]. Therefore, the main function of the protein is to provide a selective environment for the relevant substrate and insulate the active site from the solvent water so that the uncoupling that results in the generation of the harmful hydrogen peroxide can be avoided [16,20].

**Figure 3:**
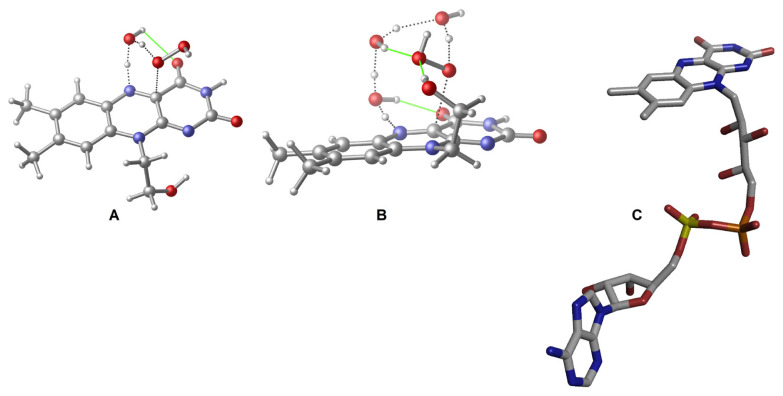
Reference TSs that involve the activation with one water molecule and three water molecules (**A** and **B**, respectively) [25]. The alignment of the isoalloxazine’s representative substituent is drastically changed in comparison to its actual alignment (**C**; H atoms are not shown), as seen in a usual monooxygenase (pdb id: 1DOD). The change in **B** is more important because it enables an additional unnatural H-bonding interaction. H-bonds are shown in green color.

According to experimental studies, deprotonation of the N_5_ atom (for the atomic labels, please see Figure 1) plays a pivotal role in the uncoupling event [37]. Based on these studies, a concerted mechanism was put forward in which the hydrogen of N_5_ forms H-bonding interactions with both hydroperoxide oxygen atoms (the one which binds to C_4a_ carbon is known as the proximal oxygen (O_p_) while the other is known as the distal oxygen (O_d_), as defined in Figure 1) in the transition state (TS) which is eventually followed by the H_2_O_2_ release. This idea was challenged by a computational study in which the authors show how a concerted mechanism would require high barrier energy [25]. Although, a more recent calculation showed that the barrier energy of a concerted mechanism for this step was approximately 25 kcal/mol, it still cannot compete with monooxygenation [11]. This is important because, as mentioned earlier, the uncoupling in a monooxygenase takes place even in the presence of a substrate. According to the gas phase calculations of the former study, local environment related interactions are necessary for H_2_O_2_ generation [25]. Naturally, water molecules were one of the investigated effectors of H_2_O_2_ generation. With the participation of one water molecule as a catalyst, the TS barrier energy required 19.51 kcal/mol, which is a feasible value for a reaction to take place. While the inclusion of a second water molecule did not change the barrier significantly, a TS structure with three water molecules lowered the barrier energy to 16.24 kcal/mol. These calculations certainly should be a step up from the previous proposition since the easy decay of C(4a)-(hydro)peroxide intermediate by itself would render it useless as a cofactor. These calculations also imply that even a single water molecule can catalyze the decay of the intermediate into its oxidized state (Fl_ox_) and an H_2_O_2_ molecule. However, as displayed in some of the X-ray structures, there is a recurring water molecule near the isoalloxazine ring system of a monooxygenase [38,39]. Moreover, this water molecule was shown to participate in the normal catalytic activity of the enzyme [20]. These calculations should be reconsidered because the remaining part of the flavin, that is bound to the isoalloxazine ring system from the N_10_ position, was modeled as an unrestrained −(CH_2_)_2_–OH moiety (Figure 3). The hydroxyl of this moiety formed an H-bond with only some of the TS structures, complicating the comparison of TSs with different numbers of water molecules (Figure 3). In its natural environment, this part of the cofactor can never change its conformation to allow these interactions (Figure 3) because it corresponds to a large moiety that is buried within the enzyme and stabilized with various residues, which restrict its rotation. In addition, finding the minimum number of water molecules that allow the reaction to evolve with a feasible barrier energy requires reconsideration of these calculations with the right solvation scheme instead of the gas phase.

In this manuscript, first, density functional theory (DFT) calculations were carried out to find the minimum number of water molecules necessary to reduce the barrier energy of uncoupling to about that of a monooxygenation when the reaction takes place at a water exposed location. Secondly, for the first time in the literature, a quantum cluster model [40] (please see computational methods for details) of the active site of kynurenine 3-monooxygenase was built to see how the uncoupling reaction takes place within the active site of the enzyme by the activation of water molecules. This study can enhance our understanding of C(4a)-(hydro)peroxyflavin stability which was described as a fundamental challenge in a recent review [2].

## 2. Computational methods

The optimizations of the usual models were carried out using Gaussian 16 Revision A.03 software package at the B3LYP/6-31+G(d,p) level of theory [41]. Each TS was confirmed to have only one imaginary frequency. Reactant and product states, which did not have any imaginary frequency apart from the product states P1v2 (7.85 i cm^–1^) and P2v2 (12.34 i cm^–1^), were located by following the points obtained from the intrinsic reaction coordinate (IRC) calculations, as implemented in Gaussian 16 [42,43]. The imaginary frequencies mentioned were ignored because they are very low and belong to the product states that do not affect the forward barrier energies. The solvent water was represented by polarizable continuum model (PCM) in all of these calculations [44]. In addition, the larger basis set 6-311+G(2d,2p) was utilized in the single point calculations to refine the electronic energies to which DFT-D3(BJ) scheme dispersion corrections [45,46] and the zero-point energy corrections that were obtained from the optimization level were added while solvent water was represented by PCM.

The active site of KMO was built starting from the X-ray structure 6FOX.pdb for the quantum cluster modeling that was also previously utilized in the mechanistic studies of KMO [20,47]. In this model, the FAD unit was mainly modeled as the isoalloxazine ring system. The remaining part of FAD was represented with a methyl moiety whose carbon atom’s coordinates were fixed during optimizations. In addition, a hydroperoxide moiety was attached to the C_4a_ position. L-Kyn substrate in the original structure was replaced with a water molecule. The β sheet below the isoalloxazine ring system was represented with the Asn54-Leu55-Ala56-Leu57 array of residues and the positions of the α carbon atoms of Asn54 and Leu57 residues, which were capped with hydrogen atoms, were fixed in their X-ray coordinates. The loop above the isoalloxazine ring system was treated in the same way and represented with the Val317-Pro318-Phe319-His320-Gly321-Gln322-Gly323-Met324 array of residues. Ile224, Leu226, Thr236, and Phe238 were represented with their side chains. Eight water molecules that were present in the active site of the X-ray structure were retained in the calculations. The neutral model is comprised of 258 atoms. Nine atoms were fixed in their X-ray positions during the geometry optimizations. The optimizations of the cluster models were carried out at the B3LYP/6-31G(d,p) level of theory. This level of theory proved to be efficient in previous computational studies for this complex [20,47]. TSs were located as explained above and similarly, the reactant and the product geometries were obtained through the optimizations of the corresponding IRC point geometries. In the single point calculations, the basis set was enlarged to 6-311+G(2d,2p), and the dispersion and the zero-point energy corrections were added to the electronic energies as described above. These single point calculations were repeated in different dielectric constants (ɛ = 1, 4, 16, 80) to reveal the competency of the size of the cluster models.

## 3. Results and discussion

Initially, the water-activated uncoupling was studied in a solvent water exposed environment. In this initial study, general conclusions relevant to any monooxygenase are sought. Therefore, the computational system was assumed to be surrounded with water, and the PCM solvation scheme was adapted accordingly to represent the reaction conditions.

In the second part of this study, the uncoupling was modeled within the active site of KMO using the quantum cluster computational scheme. This study can also reveal the importance of the predefined positions of the water molecules observed in the active site of KMO. In a previous computational study [20], it was alluded that there is a conserved H-bonding network of water molecules in the active site of KMO. This network is observed in different X-ray structures of KMO (5NAK.pdb and 6FOX.pdb) that were obtained by different experimental groups. The current study can reveal new findings about the necessity for these water molecules, which are potential reactants in the uncoupling reaction, to be in predefined positions.

C(4a)-(hydro)peroxide intermediate was modeled as the isoalloxazine ring system that is bound to the hydroperoxy moiety, and the remaining fragment was represented with a methyl moiety. As explained in the introduction, adding the remaining part of this large molecule is unnecessary since it orients further away, and it does not take part in the reaction. Additionally, the inclusion of this part causes problems when the specific enzyme environment is not introduced into the computational system.

**Figure 4:**
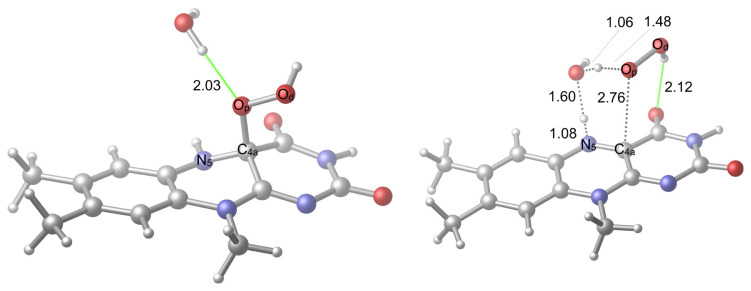
Reactant (**R1v1**) and TS (**TS1v1**) geometries related to the activation with one water molecule. H-bonds are shown in green color.

**Figure 5:**
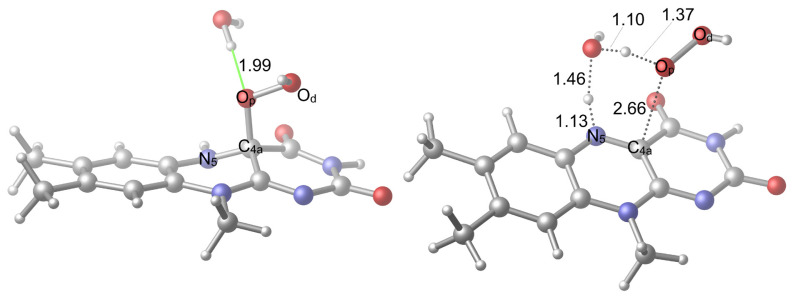
Alternative reactant (**R1v2**) and TS (**TS1v2**) geometries related to the activation with one water molecule. H-bonds are shown in green color.

Herein, we are interested in particular H-bonding possibilities which can activate the uncoupling through a proton shuttle; therefore, N_5_ position is considered in the formation of H-bonds, which are mediated by water molecules. The water molecules can interact with other positions of the isoalloxazine ring system, but these are not primary interactions that can aid the activation. Two alternative pathways were considered for the uncoupling mediated by one water molecule. These involve the transfer of the positively charged hydrogen (H^+^), which is bound to N_5_, to the oxygen atom of a water molecule whose own H^+^ is transferred to O_p_. This induces the breaking of C_4a_–O_p_ bond that liberates a free H_2_O_2_. In the first case (Figure 4), the hydroxyl hydrogen of the hydroperoxide moiety orients towards the carbonyl oxygen of the isoalloxazine ring system, where the formation of an H-bonding interaction opportunity arises in the TS structure (TS1v1); in the second case (Figure 5), the hydroxyl hydrogen of the hydroperoxide moiety orients in the opposite direction.

**Figure 6:**
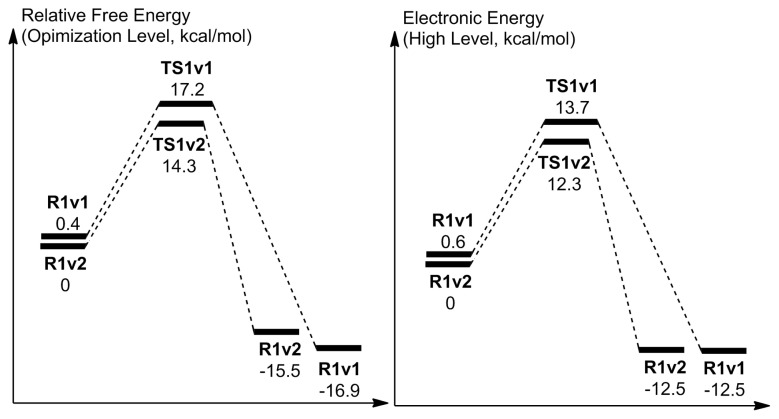
Reaction coordinate related to the activation with one water molecule. Energies are not to scale.

At first glance, it seems that a stabilization possibility was discarded with the second option. However, the first option also involves a repelling interaction due to the proximity of the mentioned hydrogen of hydroperoxide moiety to the H^+^ that is being transferred from the water oxygen to O_p_. The comparison of the reactant geometries of the two options in Figures 4 and 5, respectively, shows that they are significantly alike. The only difference is the mentioned orientation of the hydroperoxide hydrogen. This difference in the reactant geometries is not significant thermodynamically since the free energy difference (at the optimization level) between the respective structures is 0.4 kcal/mol while their electronic energy difference (at high-level) is 0.6 kcal/mol (Figure 6). According to the schemes given in Figure 6, there is 2.9 kcal/mol and 1.4 kcal/mol of a reduction in the relative TS free energy and the relative electronic energy from first to the second case, respectively. Therefore, the barrier energy differences are mainly due to the TS energies. These barrier energies that correspond to the reaction conditions when the hydroperoxide intermediate is vulnerable to the solvent water molecules render the uncoupling reaction feasible.

**Figure 7:**
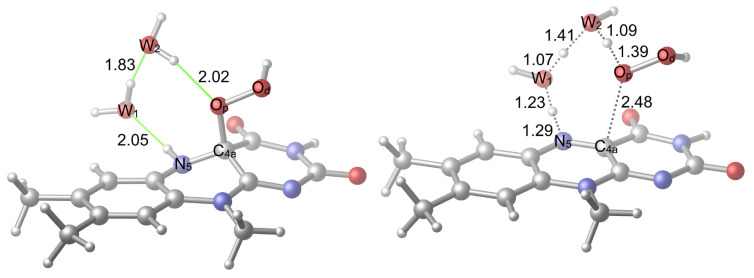
Reactant (**R2v1**) and TS (**TS2v1**) geometries related to the activation with two water molecules. H-bonds are shown in green color.

**Figure 8:**
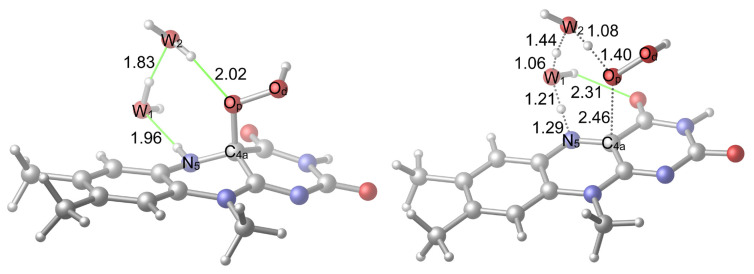
Alternative reactant (**R2v2**) and TS (**TS2v2**) geometries related to the activation with two water molecules. H-bonds are shown in green color.

The reaction was reconsidered with the addition of a second water molecule (Figure 7). Two possibilities that involve the transfer of the H^+^ bound to N_5_ to the first water (W_1_) oxygen whose own H^+^ is transferred to the second water (W_2_) oxygen were considered (the alternative is in Figure 8). The W_2_ was placed between the W_1_ and O_p_ to increase the number of the members of the proton shuttle so that it can further aid the activation of the uncoupling by decreasing the strain of the TS geometry. Other H-bonding sites were not considered because these sites would not aid the activation as they should do by increasing the size of the proton shuttle. In addition, placing the W_2_ in other H-bonding sites would not enable a TS structure similar to what is seen in Figures 7 and 8 because the W_2_ would be further away and can only influence the reaction via a weaker interaction. This proton shuttle induces the transfer of W_2_’s own H^+^ to O_p_ and the breaking of the C_4a_–O_p_ bond that liberates a free H_2_O_2_.

In the first case (Figure 7), the hydrogen of W_2_, whose H^+^ is to be transferred to the O_p_, can form an H-bonding interaction with the carbonyl oxygen of the isoalloxazine ring system. Here, the orientation of the hydrogen was made available, but the H-bonding interaction was not realized. In the second case (Figure 8), it is the hydrogen of W_1_ that can form an H-bond with carbonyl oxygen. Another difference between the two systems is that in the first case, the immobile hydrogen of W_2_ points in the same direction as the hydroperoxide hydrogen, while in the second case it points in the opposite direction.

**Figure 9:**
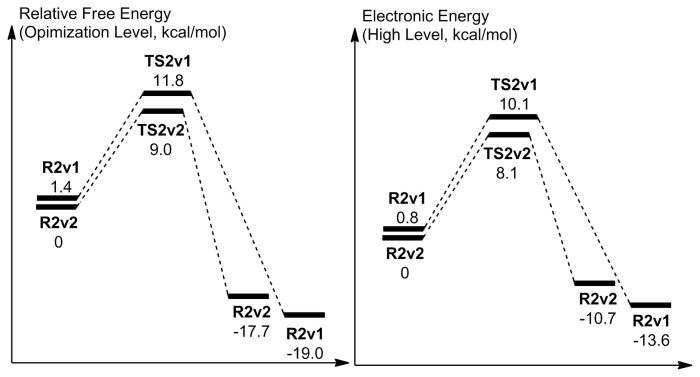
Reaction coordinate related to the activation with two water molecules. Energies are not to scale.

As can be seen in Figure 9, the relative TS free energy of the second case is 2.8 kcal/mol lower in comparison to the first case, while their electronic energy difference is 2.0 kcal/mol. The difference between the free and the electronic energies of the reactant geometries are 1.4 kcal/mol and 0.8 kcal/mol, respectively. Therefore, in the barrier energy differences (Figure 9) both the reactant and the TS geometries play a significant role.

The obtained barrier energy differences can be understood by comparing the reactant and the TS structures shown in Figures 7 and 8: In both reactant geometries, there is no H-bonding interaction apart from the H-bonding interactions that is going to involve the mobile hydrogen atoms in the corresponding TSs while, as stated, the reactant in the second case is still more stable. However, in the TS structures there is a clear difference: In the first case, there is no H-bonding interaction between the carbonyl oxygen and W_2_’s hydrogen while in the second case, there is an H-bonding interaction between W_1_’s hydrogen and the carbonyl oxygen. Moreover, the repelling interaction between W_2_’s hydrogen and the hydroperoxide hydrogen is absent in the second case. Therefore, these additional stabilizations render the second case TS-first case TS energy differences more significant in comparison to the second case reactant-first case reactant energy differences. However, both free energy and electronic energy barriers of both reactions activated by two water molecules are lower in comparison to the ones that are activated by one water molecule.

**Figure 10:**
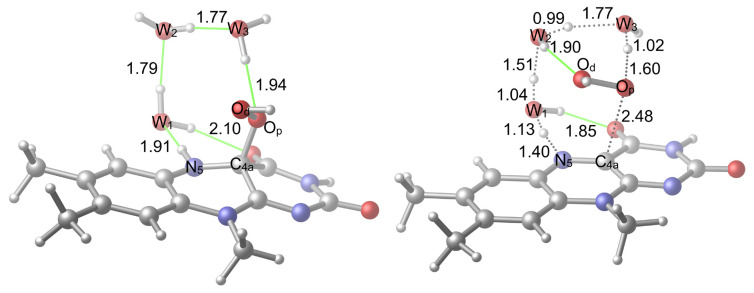
Reactant (**R3**) and TS (**TS3**) geometries related to the activation with three water molecules. H-bonds are shown in green color.

**Figure 11:**
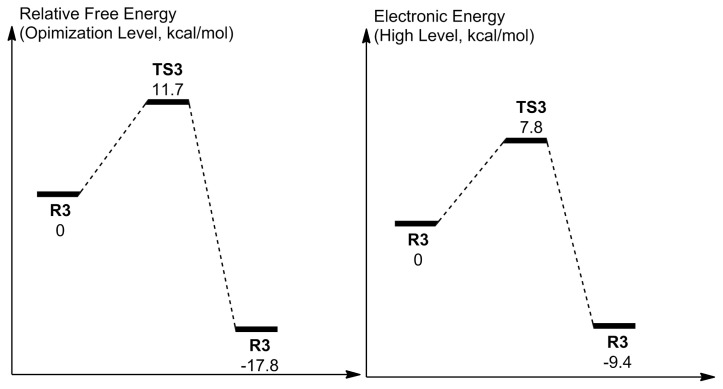
Reaction coordinate related to the activation with three water molecules. Energies are not to scale.

The mechanism of uncoupling with three water molecules (Figure 10) involves the simultaneous displacement of positively charged hydrogen centers (4 H^+^s) from N_5_ to O_p_ direction, through the mediation of three water molecules. This eventually breaks the C_4a_–O_p_ bond and liberates a free H_2_O_2_. The free energy barrier is 11.7 kcal/mol while the electronic energy barrier is 7.8 kcal/mol (Figure 11). Accordingly, the reaction with three water molecules proceeds with a higher free energy barrier while the electronic barrier energy is roughly the same in comparison to the reaction with two water molecules. Therefore, increasing the number of water molecules that mediate the uncoupling reaction is not required, and the reaction activated by two water molecules can be considered the main reaction when the system is exposed to the solvent water.

**Figure 12:**
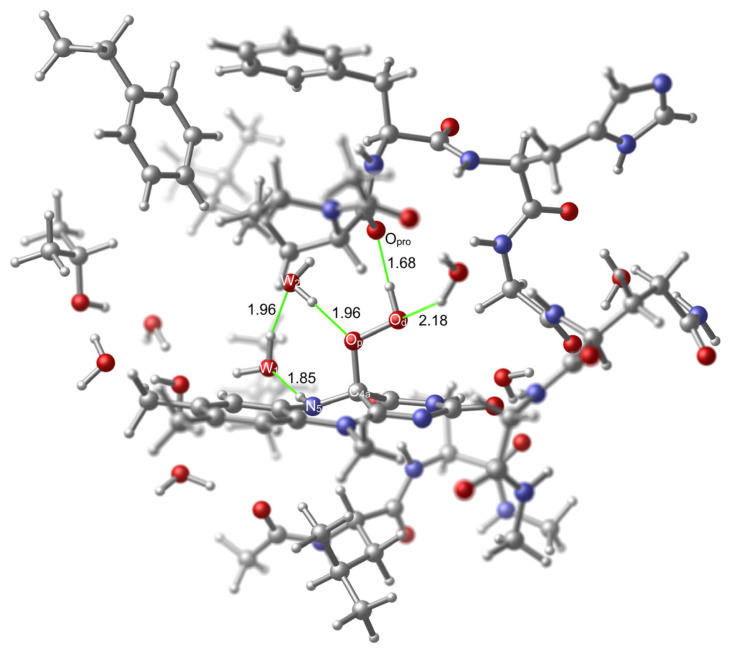
Reactant (**RC**) geometry related to the activation within the active site of KMO. Few important H-bonds are shown in green color. Several atoms were left out of focus to make the involved portion of the model apparent.

In the cluster model calculations, just like in a previous computational study [20], the two water oxygen atoms were used to generate the hydroperoxide moiety of the model of C(4a)-(hydro)peroxide intermediate. Their positions allowed modeling this moiety in a way similar to that is seen in the structure R2v1. The original positions of these oxygen atoms are suitable to obtain the desired conformational state of the hydroperoxide moiety because the one that corresponds to the proximal oxygen (which is required to interact with the water hydrogen) is closer to the water hydrogen in comparison to the one that corresponds to the distal oxygen (Figure 12). In this model, the first water molecule (W_1_) that is involved in the mediation of the transformation is connected to the H-bonding network that extends with several other water molecules towards the Thr236 hydroxyl (Figure 12). As explained in the methods section, there is one additional water molecule (W_2_) that takes the place of the substrate that was present in the original X-ray structure. This new water molecule and the first water molecule that is connected to the H-bonding network were used as the mediating agents in the formation of the TS.

The active site of the KMO is extremely restrictive. For example, it does not allow a TS model as seen in TS2v2 because if the orientation of the immobile hydrogen of the second water molecule is as in the TS2v2, it causes steric clashes with the hydrogen atoms that are attached to the ring of Pro318. In addition, the removal of L-Kyn from the active site was strictly necessary as it blocks the formation of the H-bonding network that is seen in R1v1 or R2v1. Therefore, the sealing effect of the substrate that was previously alluded [47] is once again reinforced in this study. This means that if the substrate is within the active site of the enzyme while the uncoupling reaction is taking place, its position must be altered in a way that it should not block the formation of an H-bonding interaction like the one seen in R2v1. A TS structure that was mediated by three water molecules is not necessary as described above, since this results in a higher barrier. Even if it were not the case, the formation of a TS structure like TS3 would not be possible as the residues in the loop above the isoalloxazine ring system clash with the mobile atoms of such TS.

As can be seen in Figure 12, one water molecule that originates from the X-ray structure stabilizes the distal oxygen atom with an H-bonding interaction while the hydrogen that is bound to the distal oxygen atom is stabilized by the H-bonding interaction that it forms with the oxygen atom of Pro318’s carbonyl (O_pro_). Stabilization with the O_pro_ is a conserved trait in the monooxygenation reaction [20]. Therefore, adopting a TS geometry that benefits from this interaction is suitable in the uncoupling reaction as well.

**Figure 13:**
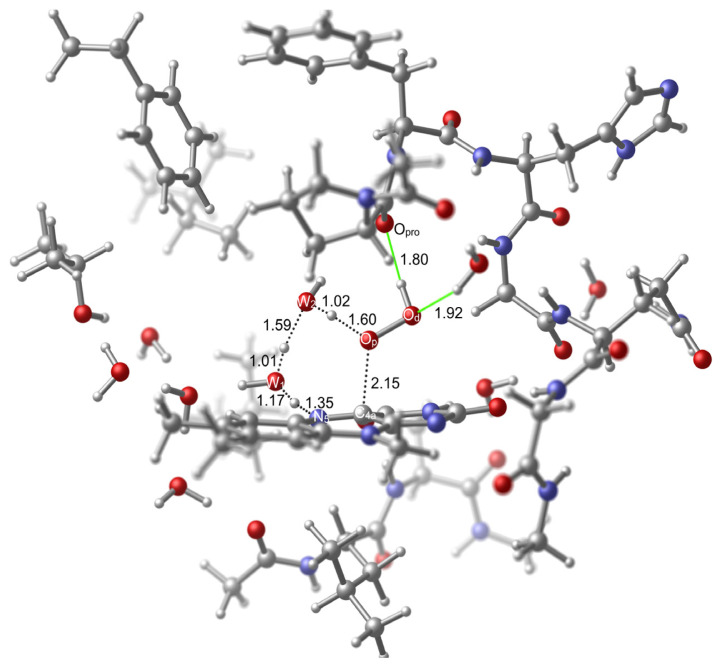
TS (**TSC**) geometry related to the activation within the active site of KMO. Few important H-bonds are shown in green color. Several atoms were left out of focus to make the involved portion of the model apparent.

As can be seen in Figure 13, in the TS structure, many distances are roughly retained in comparison to those in the model TS (TS2v1 in Figure 7). The most apparent variation in the distances is seen in the C_4a_–O_p_ separation which is shortened by 0.33 Å in comparison to that in TS2v1. This contraction is compensated with the increase in the reaction path distances (dotted lines) between the W_1_ oxygen and the W_2_ oxygen (0.12 Å), and the W_2_ oxygen and the proximal oxygen (0.14 Å). There is no variation in the reaction path distance between the N_5_ and the W_1_ oxygen. The stabilizations obtained by the H-bonding interactions between the hydroperoxide moiety and the O_pro_ or the nearby water hydrogen are retained to a great extent in comparison to those in the reactant structure (RC), since the O_pro_ – hydroperoxide hydrogen, and the O_d_ – the water hydrogen distances vary slightly, 0.12 Å and −0.26 Å, respectively, and in opposite directions.

**Figure 14:**
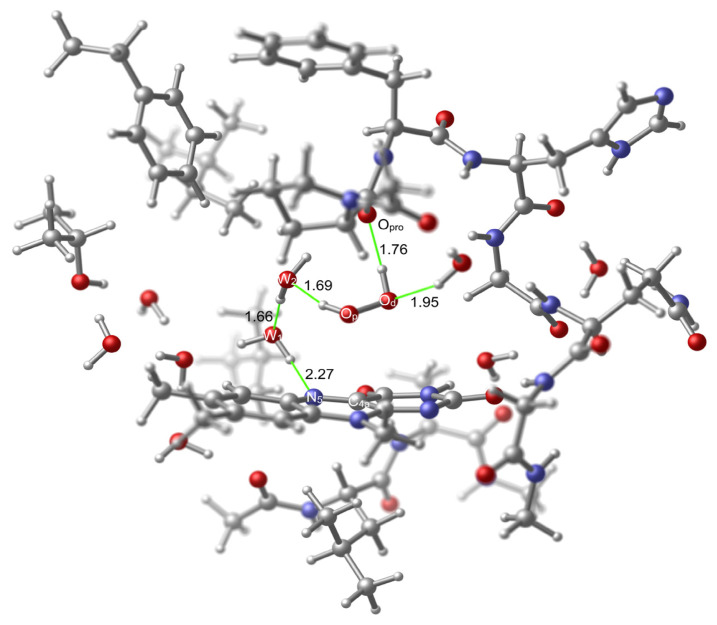
Product (**PC**) geometry related to the activation within the active site of KMO. Few important H-bonds are shown in green color. Several atoms were left out of focus to make the involved portion of the model apparent.

Formation of the H_2_O_2_ is completed in the product state of the cluster model (PC in Figure 14) where H_2_O_2_ is stabilized through several H-bonding interactions within the active site, and the model FAD returns to its original oxidation state.

**Figure 15:**
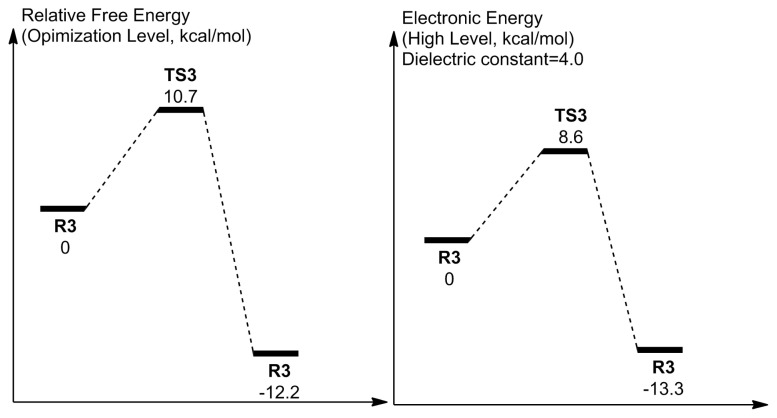
Reaction coordinate related to uncoupling within the active site of KMO. Energies are not to scale.

The conservation of the mentioned geometrical features in TSC in comparison to both TS2v1 and RC is reflected in the thermochemistry of the reaction as can be seen by comparing the energies presented in Figures 9 and 15. The free energy barrier of the cluster model changes 0.3 kcal/mol while the electronic energy barrier changes −0.7 kcal/mol in comparison to the corresponding barriers seen in Figure 9 for the first case. Here, we stated the electronic energies when ɛ = 4.0 but as it will become clear in the following discussion, the variation of the dielectric constant does not affect the reaction thermochemistry, which reveals the competency of the size of the cluster model.

The comparison of the high-level electronic energy barrier (with said corrections at ɛ = 4.0) of the uncoupling to that of the monooxygenation of L-Kyn allows the revelation about the reaction site inhibitory capability against the uncoupling reaction. According to previous two computational studies in which the same cluster approach scheme was utilized, the high-level electronic energy barrier (with said corrections at ɛ = 4.0) of the monooxygenation was found as 19.7 kcal/mol or 16.2 kcal/mol depending on the conformational state of the starting X-ray structure [20,47]. These values are clearly higher than the one (8.6 kcal/mol in Figure 15) found for the uncoupling reaction in this study. According to these values, if the overall enzyme architecture occasionally allows the permeation of water molecules into the reaction site with a configuration as described for the cluster models, the uncoupling that has a lower barrier height can take place instead of the monooxygenation of L-Kyn. The active site has the ability to block the water molecules most of the time, since the production of 3-HK by the monooxygenation of L-Kyn is an experimentally established fact. Another condition for the uncoupling to take place within the active site is the movement of L-Kyn away from the reaction site. Insulation is the main defense mechanism of KMO against the decay of C(4a)-(hydro)peroxide by uncoupling since the reaction site architecture allows uncoupling. This insulation also includes the water molecules being at the predefined positions within the active site, and the blocking effect of the L-Kyn.

The reduction of FAD’s isoalloxazine ring system by NADPH was hypothesized to take place at an exterior location but the corresponding short-lived conformational state has never been observed [29]. According to this hypothesis, after the reduction takes place, the conformational state of FAD changes instantly and the reduced isoalloxazine ring system returns to its original location in the active site. Although uncoupling at an exterior location is energetically feasible by itself, the fact that it depends on an unstable conformational state must be noted. Uncoupling at an external location also requires the rapid oxygenation [35] to take place therein so that the reactive hydroperoxide intermediate can form.

According to Table, the change of the dielectric constant does not strongly influence the relative energy differences. The most apparent deviation was seen in the barrier energy of the ɛ = 1.0 solvation scheme. The other values are almost equivalent. Even the barrier energy of the ɛ = 1.0 solvation scheme deviates only about 1 kcal/mol relative to the barrier energies of other solvation schemes. These results clearly validate the competency of the size of the computational system.

## 4. Conclusion

In this manuscript, the formation of free hydrogen peroxide due to the decay of the reactive C(4a)-(hydro)peroxide via uncoupling was investigated. The reaction was considered in two different environments: First, when the reaction system is exposed to solvent water; and secondly, when the reaction system is isolated from the solvent water within the active site of an enzyme, KMO. According to the results of the first part, the reaction is mediated by two water molecules in the working mechanism, since the reaction activated by one water molecule results in higher barrier energies while the reaction activated by three water molecules results in higher free energy barrier and roughly the same high-level electronic energy barrier. Nonetheless, all three mechanistic pathways are feasible; therefore, when the reaction system is exposed to solvent water, each of them can take place to some extent. According to the second part of this study in which the quantum cluster approach was utilized in modeling the uncoupling reaction within the active site of KMO complex that was represented with 258 atoms, the reaction site of KMO does not inhibit the uncoupling because the barrier energies were found to be very similar to that of the model with two water molecules that is exposed to solvent water. Therefore, the reaction is feasible. The barrier energy of the uncoupling, being almost half of that of the monooxygenation of L-Kyn, renders it inevitable once a water molecule enters into the active site and the substrate L-Kyn (which blocks the formation of any kind of uncoupling TS studied in this manuscript) is moved away. These results imply that the observed hydrogen peroxide formation by uncoupling is either due to the permeation of water molecules into the reaction site or the formation of the hydroperoxide intermediate at an external location of the enzyme. However, it must be noted that the duration of the conformational state of FAD that allows it to be at an external location must be extremely short, since this conformational state has never been observed [29,35].

These results strengthen the thesis that the main defense mechanism of a monooxygenase is insulation. The insulation of KMO also includes the blocking of the water molecules by the substrate L-Kyn itself which seals the entrance to the reaction site as seen in the X-ray structures. The presence of the water molecules at predefined positions [20], as seen within the active sites of different KMO–L-Kyn X-ray structures, must be due to the insulation mechanism because these water molecules are potential catalysts in the uncoupling reaction.

## Figures and Tables

**Table:** High-level electronic energies (kcal/mol) with respect to different dielectric constants. The energies are calculated relative to the reactant state (**RC**) in each solvation scheme.

State	ϵ = 1	ϵ = 4	ϵ = 16	ϵ = 80
**RC**	0	0	0	0
**TSC**	9.6	8.6	8.6	8.8
**PC**	−12.9	−13.3	−13.3	−13.6
